# Quantitative perfusion assessment using indocyanine green during surgery — current applications and recommendations for future use

**DOI:** 10.1007/s00423-023-02780-0

**Published:** 2023-01-26

**Authors:** P. Van Den Hoven, J. Osterkamp, N. Nerup, M. B. S. Svendsen, Alexander Vahrmeijer, J. R. Van Der Vorst, M. P. Achiam

**Affiliations:** 1 Department of Surgery, Leiden University Medical Center, Leiden, The Netherlands; 2 Department of Surgery and Transplantation, Copenhagen University Hospital Rigshospitalet, The Capital Region of Denmark, Copenhagen, Denmark; 3 CAMES Engineering, Copenhagen Academy for Medical Education and Simulation, Centre for Human Resources and Education, The Capital Region of Denmark, Copenhagen, Denmark

**Keywords:** Near-infrared fluorescence imaging, Indocyanine green, Quantification, Perfusion

## Abstract

**Purpose:**

Incorrect assessment of tissue perfusion carries a significant risk of complications in surgery. The use of near-infrared (NIR) fluorescence imaging with Indocyanine Green (ICG) presents a possible solution. However, only through quantification of the fluorescence signal can an objective and reproducible evaluation of tissue perfusion be obtained. This narrative review aims to provide an overview of the available quantification methods for perfusion assessment using ICG NIR fluorescence imaging and to present an overview of current clinically utilized software implementations.

**Methods:**

PubMed was searched for clinical studies on the quantification of ICG NIR fluorescence imaging to assess tissue perfusion. Data on the utilized camera systems and performed methods of quantification were collected.

**Results:**

Eleven software programs for quantifying tissue perfusion using ICG NIR fluorescence imaging were identified. Five of the 11 programs have been described in three or more clinical studies, including Flow® 800, ROIs Software, IC Calc, SPY-Q™, and the Quest Research Framework®. In addition, applying normalization to fluorescence intensity analysis was described for two software programs.

**Conclusion:**

Several systems or software solutions provide a quantification of ICG fluorescence; however, intraoperative applications are scarce and quantification methods vary abundantly. In the widespread search for reliable quantification of perfusion with ICG NIR fluorescence imaging, standardization of quantification methods and data acquisition is essential.

**Supplementary Information:**

The online version contains supplementary material available at 10.1007/s00423-023-02780-0.

## Introduction

Surgeons rely on physical examination and inspection for perfusion assessment. Although clinical observations are vital, a non-invasive, objective, and reliable instrument to support clinical decision-making would be advantageous [1, 2]. For example, within gastrointestinal surgery, it has been reported that subjective perfusion assessment lacks predictive accuracy for anastomotic leakage after intestinal resection [3]. In the search for objective perfusion assessment, several techniques have been examined, including but not limited to hyperspectral imaging, computed tomography, and laser speckle contrast imaging [4–7]. Near-infrared (NIR) fluorescence imaging using indocyanine green (ICG) is a technique gaining popularity for tissue perfusion assessment. This non-invasive imaging technique consists of a light source to excite the target tissue, combined with a camera measuring returning fluorescence intensity [8]. Imaging in the NIR spectrum has an advantage over visible light due to a relatively deep tissue penetration of up to 1 cm and the low effect of autofluorescence [9]. The usability of ICG for NIR imaging is primarily the excellent fluorescence properties in the NIR light spectrum and the binding to plasma proteins following intravenous administration, making it feasible for perfusion assessment [10]. Since the first introduction of ICG for angiography in ophthalmology, the technique has been adopted by various surgical fields, including but not limited to vascular-, gastrointestinal-, cardiac-, and reconstructive surgery [11–17]. Within vascular surgery, ICG NIR fluorescence imaging has been reported to potentially predict clinical outcomes and guide treatment strategies [18–21]. In gastrointestinal surgery, tissue perfusion assessment using ICG NIR fluorescence imaging has several promising indications, including anastomosis perfusion evaluation to identify patients at risk for anastomotic leakage [22–24]. Other surgical fields in which the prediction of tissue viability can improve patient outcome include reconstructive-, endocrine- and transplant surgery [25–27]. However, despite the widespread use of ICG NIR fluorescence imaging in clinical practice, there is still debate about the interpretation of the observed fluorescence intensity. Several clinical studies have been performed demonstrating visual (i.e., qualitative) ICG NIR perfusion assessment to be able to reduce the risk of, for example, anastomotic leakage [28]. However, the high heterogeneity among these studies and the lack of large randomized controlled trials hamper fierce statements on the value of qualitative ICG. Furthermore, outcomes on several of these phase III studies have yet to be finalized and published [29, 30]. The increased interest in quantitative perfusion assessment can be explained by the fact that a visual interpretation is subjective and inhibits the establishment of perfusion cutoff values. It has also been shown that a visual interpretation might lead to an incorrect understanding of tissue perfusion [31, 32]. In recent years, both clinical—and preclinical studies on quantification of the fluorescence intensity are increasingly being performed using specifically designed software programs [33, 34]. However, there is ongoing discussion on the most reliable method of quantitative perfusion assessment precluding standardized use in clinical practice. Despite this lack of reliability, however, there is a rapidly growing use of commercially available systems for quantification of ICG NIR fluorescence imaging. Therefore, this review aims to provide an overview of the various quantification methods for ICG NIR fluorescence imaging described in clinical studies with recommendations for future development.

## Material and methods

PubMed was searched for clinical studies on quantitative tissue perfusion assessment using ICG NIR fluorescence imaging published before January 2022. Studies lacking a description of the camera system or utilized software program were excluded. The search strategy (Appendix A) identified 199 articles of which 16 were eligible for use, alongside 48 articles published in an earlier review by our study group [34]. The following features concerning the quantitative perfusion assssment were identified: (1) software program, (2) the camera system, (3) the surgical specialty of use, and (4) featured quantification method(s).

### Quantification

**Fig. 1:**
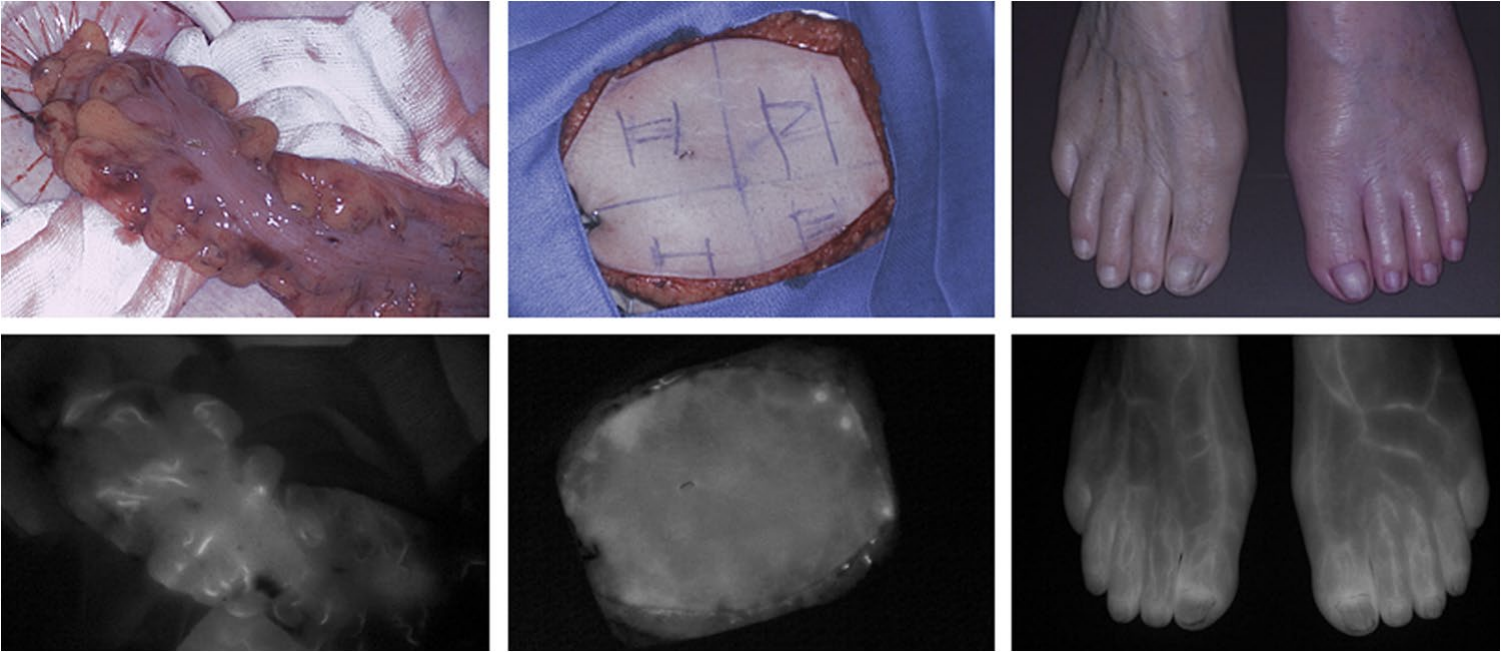
ICG NIR fluorescence imaging using the Quest Spectrum Platform® showing the visual (above) and near-infrared fluorescence (below) output for three potential indications. Left: intraoperative perfusion assessment of the descending colon in a 65-year old female patient undergoing a low anterior resection. Middle: intraoperative perfusion assessment of a profunda artery perforator flap in reconstructive breast surgery of a 38-year old female patient. Right: postoperative perfusion assessment of a 73-year old female patient following peripheral arterial bypass surgery on the left side

**Fig. 2:**
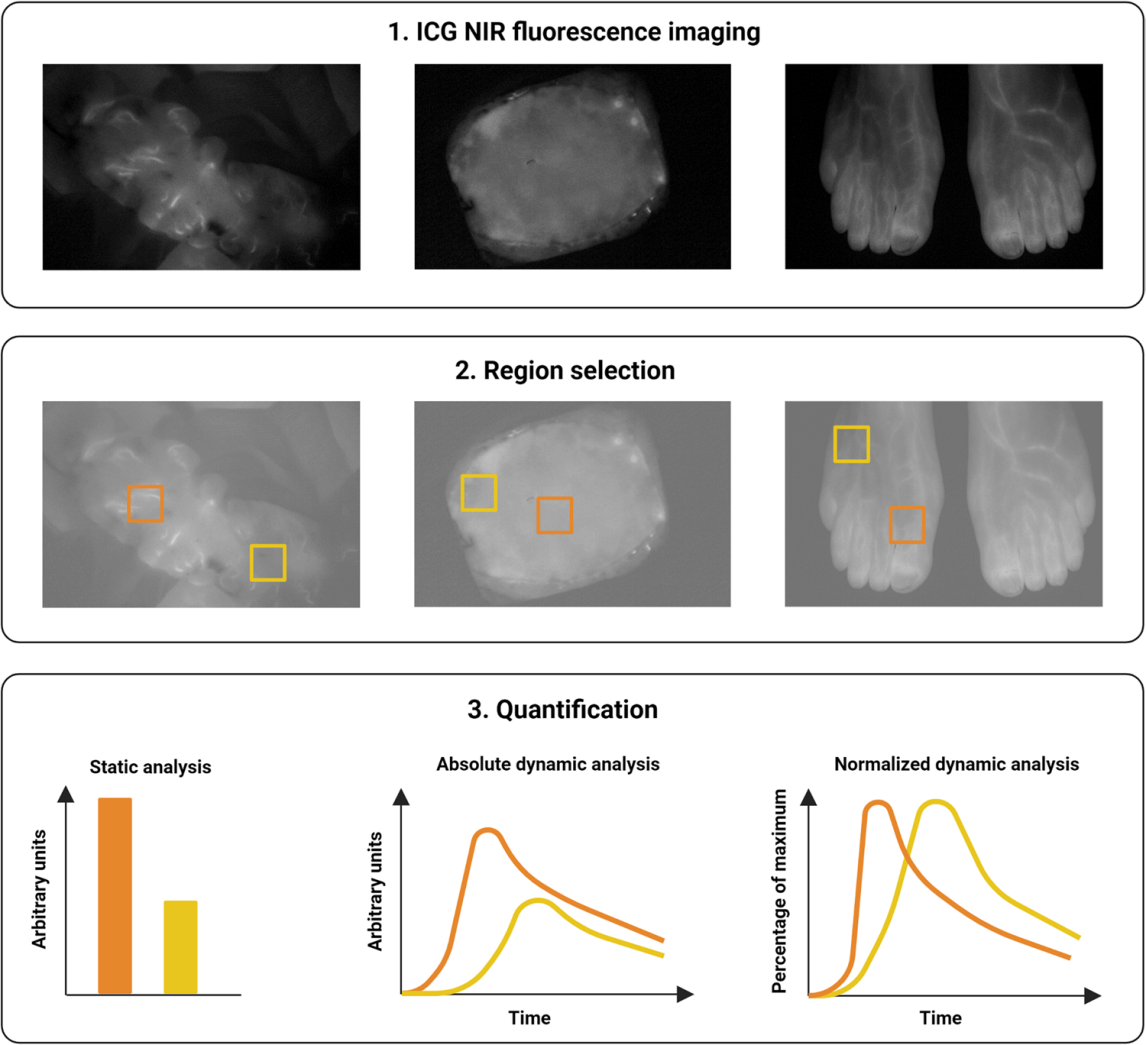
Standardized work flow for quantitative perfusion assessment using near-infrared fluorescence imaging with indocyanine green. First, ICG NIR fluorescence imaging is performed creating a fluorescence intensity image or video. Secondly, the target area or region of interest (ROI) is selected using specifically designed software programs for quantitative analysis of the fluorescence intensity. The third and final step of the quantification workflow is the actual quantitative analysis of the fluorescence intensity which can be performed using three methods: (1) static analysis (i.e., point analysis without dynamics over time), (2) absolute dynamic analysis by using so called time-intensity curves, and (3) normalized dynamic analysis, i.e., describing the fluorescence intensity over time as a percentage relative to the maximum intensity. *Created with BioRender*

Quantitative perfusion assessment with ICG NIR fluorescence imaging is performed in three steps. First, an ICG NIR fluorescence imaging camera system visualizes the fluorescence intensity in the camera’s field of view, producing a grayscale image or video (Fig. 1 and Video 1). Secondly, the target area for quantification, the region of interest (ROI), is selected. Following selection of one or multiple ROIs, the measured fluorescence intensity, defined in arbitrary units (AU), is quantified by specifically designed software programs. In short, three quantification methods can be distinguished and subdivided into (1) static fluorescence analysis, (2) dynamic absolute fluorescence analysis, and (3) dynamic normalized fluorescence analysis (Fig. 2).

1. Static analysis is performed by measuring the absolute intensity in an ROI in a single point in time, either displayed in AU or as a percentage relative to a reference point [16, 35, 36]. The absolute or relative perfusion units can be displayed in the image or visualized in a color-coded heat map.
2. In dynamic absolute fluorescence analysis, the intensity is described as the change in AU over time, most often using a time-intensity curve. Of these curves, several parameters including in- and outflow values can be extracted, which have been outlined in previous review articles on quantification of tissue perfusion with ICG NIR fluorescence imaging [24, 33, 34].
3. In dynamic normalized fluorescence analysis, the fluorescence intensity is adjusted for the maximum intensity (i.e., normalized) in the selected ROI [37, 38]. This method describes the fluorescence intensity as a percentual change of the maximum intensity over time rather than an absolute intensity change.

## Results

The PubMed search identified 11 software programs for quantifying tissue perfusion with ICG NIR fluorescence imaging. Of these 11 programs, five have been described in three or more clinical studies, including Flow® 800, ROIs Software, IC Calc, SPY-Q™, and the Quest Research Framework®. An overview of currently used quantification software for ICG NIR fluorescence perfusion assessment is presented in Table 1.

**Table 1:** Overview of ICG NIR fluorescence quantification software used in clinical studies with featured quantification methods

Specifications		References	Specialty of use	Quantification method			Intraoperative use
Software program	Utilized camera system			Static analysis	Dynamic absolute analysis	Dynamic normalized analysis	
VISIONSENSE® (Medtronic, Minneapolis, MN, USA)	EleVision™ IR platform (Medtronic, Minneapolis, MN, USA)	[39]	Endocrine surgery	Yes	NS	NS	Yes
FLER	D-light P system (Karl Storz)	[40]	Gastrointestinal surgery	NS	Yes	NS	Yes
Flow® 800 (Zeiss Meditec, Oberkochen, Germany)	Pentero operative microscope (Carl Zeiss, Oberkochen, Germany) Kinevo operative microscope (Carl Zeiss, Oberkochen, Germany)	[41–54]	Neurosurgery	NS	Yes	NS	Yes
ROIs software (Hamamatsu Photonics K.K., Hamamatsu, Japan)	D-light P system (Karl Storz, Tuttlingen, Germany), Olympus (Tokyo, Japan), PDE (Hamamatsu Photonics K. K., Hamamatsu, Japan), PDE-neo System (Hamamatsu Photonics K.K., Hamamatsu, Japan)	[55–65]	Gastrointestinal surgery Breast surgery Vascular surgery	NS	Yes	NS	Yes
IC-Calc	IC View (Pulsion Medical systems AG), CCD camera (Karl Storz Tuttlingen, Germany)	[66–71]	Reconstructive surgery Endocrine surgery Neurosurgery	NS	Yes	NS	Yes
ImageJ (National Institutes of Health, Maryland, USA), ImageJ (National Institute of Health, Bethesda, MD)	NIR laparoscope (Olympus Corporation, Tokyo, Japan), ICG camera (Pulsion Photodynamic Eye, Pulsion Medical Systems SE, Feldkirchen, Germany) Fluobeam® (Fluoptics, Grenoble, France)	[72–74]	Gastrointestinal surgery Reconstructive surgery	NS	Yes	NS	Yes
NIRx NAVI	DYNOT 232 optical tomography system (NIRx Medical Technologies LLC, NY, USA)	[75]	Breast surgery	NS	Yes	NS	NS
Q-ICG	ICG Hopkins Telescope 30° (Image-1 SPIES, D-Light P, Karl Storz GmbH and Co. KG, Tuttlingen, Germany)	[32]	Gastrointestinal surgery	NS	Yes	Yes	Yes
Quest Research Framework®	Quest Spectrum platform ® (Quest Medical Imaging, Middenmeer, the Netherlands)	[18, 26, 38, 76]	Vascular surgery Endocrine surgery	No	Yes	Yes	No
Spy-Q™	SPY Elite Imaging System (Novadaq Technologies, Ontario, Canada)	[20, 27, 77–92]	Reconstructive surgery Vascular surgery Breast surgery Transplantation surgery	Yes	Yes	NS	Yes
Tracker 4.97 (Douglas Brown, Open Source Physics, Boston MA, USA)	IMAGE1 S™, Karl Storz, Germany)	[93]	Gastroenterology	NS	Yes	NS	NS

*NS*, not specified

### Static fluorescence analysis

Most clinical studies in which static analysis was used were performed with the proprietary quantification software SPY-Q™ [77, 78]. This software program is compatible with the SPY Elite fluorescence imaging system, which features real-time intraoperative visualization of quantification. Upon manual selection of a reference region, Newman et al. described significant correlation with mastectomy skin necrosis following mastectomy using a relative perfusion value of 25.2% [77]. Similar results were obtained in a study by Moyer et al., demonstrating a cutoff of 25% to increase the likelihood of mastecomy skin flap necrosis [78]. Static analysis has also been investigated with VISIONSENSE®, a relatively new program allowing perfusion imaging laparoscopically using color-coded heat maps with a near-infrared overlay. An adjustable threshold for relative perfusion value makes it possible to visualize perfusion differences. In a recent study, Kamada et al. proved the system to be feasible in differentiating thyroid and parathyroid tissue using a relative perfusion value of 50% [94]. Although cutoff values for static analysis were found to be significant within these studies, no phase III studies have been identified for perfusion assessment with ICG NIR fluorescence imaging for intraoperative decision-making.

### Dynamic fluorescence analysis—absolute intensity

Dynamic fluorescence analysis using absolute intensity was the most reported method for quantifying ICG NIR fluorescence imaging. Of the 11 described software programs, ten are able to describe the change in absolute fluorescence intensity over time. Of these, five have been described in three or more clinical studies, including Flow® 800, ROIs Software, IC Calc, SPY-Q™, and the Quest Research Framework® (Table 1). The Flow® 800, solely used in neurosurgery, allows for intraoperative visualization of time-intensity curves in up to eight regions of interest [95]. The program also features the visualization of a color-coded map describing the start of the fluorescence intensity increase, a semi-quantitative analysis [41]. Clear map visualization is enhanced using motion tracking assistance [95]. The most reported software program for dynamic absolute fluorescence analysis was SPY-Q™. This intra-operatively usable software system provides both absolute in- and outflow parameters and has an automatic baseline fluorescence adjustment [20]. Results on dynamic analysis can be readily displayed in relative values, providing cutoff values for perfusion [79]. Another program allowing the intraoperative quantification of dynamic fluorescence analysis is ROIs software. This software program by Hamamatsu Photonics has been proven compatible with various camera systems (Table 1). Intraoperative selection of a region of interest generates absolute time-intensity curves [55]. As for static analysis, studies beyond the scope of a pilot setting are yet to be performed.

### Dynamic fluorescence analysis—normalization

The feature of applying normalization to the dynamic analysis was described by two software programs: Q-ICG and the Quest Research Framework®. Of these two techniques, Q-ICG is the only one that enables real-time intraoperative analysis [32]. This algorithm has a built-in quality control that allows surgeons to disregard information from regions with poor data quality. The system is compatible with videos in various file types and any imaging system, as a plug and play solution [32]. The Quest Research Framework® program, which is proprietary software to the Quest Spectrum platform®, enables the generation of normalized time-intensity curves [76]. This program has a built-in motion tracker adjusting for movement, limiting influence of target area movement in the horizontal plane. As with absolute fluorescence analysis, no studies have been performed assessing the value of normalization for perfusion assessment in advanced stage clinical trials.

## Discussion

This narrative review demonstrates the variety of featured quantification methods for ICG NIR fluorescence imaging within the currently described software programs. Interestingly, none of these 11 software programs can apply all three methods of quantification, despite the demonstrated potential of all three quantification methods towards clinical implentation [16, 25, 33, 34, 36, 96]. However, there is a lack of studies exploring the use of currently avaliable quantification software programs in studies beyond the scope of feasability. This might be due to the limitations associated with each quantification method. Static fluorescence analysis, for example, is sensitive to several factors impeding its reliability. First of all, the measured fluorescence intensity is subject to camera- and patient-related settings [97, 98]. Hence, the differences in camera distance and angle to the target area influence the measured intensity. Second, in case of relative static fluorescence analysis, the chosen reference point significantly influences the effect on other ROIs. This might, for example, lead to relative perfusion units above 100% [25]. Third, the timing of analysis following ICG administration can lead to misinterpretation, since it is known that ICG will also diffuse into ischemic tissue over time [40]. Dynamic absolute fluorescence analysis overcomes this time-related issue by describing the intensity change over time. However, with absolute intensity, there is still an ongoing debate on which parameter is most appropriate [34, 96]. This could partly be explained by influencing factors, including camera distance and angle, compromising the reliability of using absolute perfusion units [97]. Furthermore, as outlined in an earlier review on the quantification of ICG in gastrointestinal surgery, camera-specific characteristics will influence the measured fluorescence intensity [99]. Hence illumination, NIR spectrum bandwidth and filters, optical settings, including exposure time- and gain influence the signal [9]. Normalization tends to minimize the effects of patient- and camera-related settings by adjusting the fluorescence for the maximum intensity [100]. Although normalization enhances reliability and validity by reducing influencing factors, intensity-related parameters are depleted. These absolute parameters, such as maximum intensity, could be useful in the assessment of tissue viability, as was shown in several studies [18, 72, 101]. Furthermore, normalization of time-intensity curves in areas with low fluorescence intensity (for example within tissue necrosis), i.e., magnifying the signal to 100%, might lead to fluttering of the curves, making them non-interpretable. In the quest for developing a reliable and clinicially usable quantification system, these limitations as well as the advantages associated with each quantification method should be carefully addressed (Table 2). A recently published review highlighted the significance of a clear guideline for fluorescence imaging to be valuable for implementation in clinical practice [102]. Reliable quantification of the fluorescence intensity faces the same challenges and mandates a clear guideline. The rapidly emerging field of quantitative perfusion assessment with ICG is increasingly becoming multidisciplinary teamwork, including physicians, technicians, and commercial developers. In the quest for reliable quantification, it is pivotal that all involved parties focus in the same direction, that is, towards clinical implementation. To reach this goal, it is of utmost importance that data on fluorescence intensity is findable, accessible, interoperable, and reusable, according to FAIR principles [103]. The use of a standardized method for quantification of tissue perfusion with ICG NIR fluorescence imaging then marks a starting point towards necessary clinical trials.

**Table 2:** Three methods of quantification with recommendations for use in clinical practice

Quantification method	Advantage(s)	Disadvantage(s)	Preferred indication
Static analysis	- Easy to perform	- Incorrect timing leads to misinterpretation - Sensitive to factors influencing intensity	- None
Absolute dynamic analysis	- Information about in- and outflow	- Sensitive to factors influencing intensity - Subject to variations among arbitrary unit scale in camera systems	- Tissue viability
Normalized dynamic analysis	- Information about in- and outflow	- Inaccuracy with low intensity	- Perfusion assessment

## Conclusion

Currently utilized software programs for the quantification of ICG NIR fluorescence imaging vary in their methods of quantification and usability. In the widespread search for reliable quantification of perfusion with ICG NIR fluorescence imaging, standardization of quantification methods and data acquisition is essential.

### Supplementary Information

Below is the link to the electronic supplementary material.

Supplementary file1 (DOCX 14 KB)

Supplementary file2 Video 1 Accelerated ICG NIR fluorescence intensity video of the feet of an 88-year old male patient following intravenous administration of 0.1mg per kg ICG. Imaging was performed using the Quest Spectrum Platform®. (MP4 24155 KB)
