# Supplementary material for: Quantitative perfusion assessment using indocyanine green during surgery — current applications and recommendations for future use
Source: Langenbecks Arch Surg. 2023 Jan 26;408(1):67. doi: 10.1007/s00423-023-02780-0 (PMC9879827; doi:10.1007/s00423-023-02780-0)
Supplement: Supplementary file 1 — Supplementary file1 (DOCX 14 KB) [file 423_2023_2780_MOESM1_ESM.docx]

**Appendix A. Search strategy**

((perfusion[MeSH Terms] OR perfusion[Title/Abstract]) AND (indocyanine green[MeSH Terms] OR indocyanine green[Title/Abstract] OR ICG [Title/Abstract]) AND (quantification[Title/Abstract] OR quantitative[Title/Abstract] OR parameter [Title/Abstract]))

*Article information*

**Quantitative perfusion assessment using indocyanine green during surgery – current applications and recommendations for future use**

Van Den Hoven P ^a^, Osterkamp J ^b^, Nerup N ^b^, Svendsen MBS ^c^, Van Der Vorst JR ^a^ , Achiam MP ^b^

1. Department of Surgery, Leiden University Medical Center, Leiden, The Netherlands
2. Department of Surgery and Transplantation, Copenhagen University Hospital Rigshospitalet, The Capital region of Denmark, Copenhagen, Denmark
3. CAMES Engineering, Copenhagen Academy for Medical Education and Simulation, Centre for Human Resources and Education, The Capital region of Denmark, Copenhagen, Denmark

*Corresponding author*

P. van den Hoven, MD, PhD

p.van_den_hoven@lumc.nl
